# Editorial: Transporters: a hitchhiker’s guide to physiology, toxicology, and pharmacology—TransportDays 2022

**DOI:** 10.3389/fphar.2024.1365106

**Published:** 2024-01-15

**Authors:** Giuliano Ciarimboli, Rosalinde Masereeuw, Stefan Oswald

**Affiliations:** ^1^ Experimental Nephrology, Medicine Clinic D, Münster University Hospital, Münster, Germany; ^2^ Division of Pharmacology, Department of Pharmaceutical Sciences, Faculty of Sciences, Utrecht Institute for Pharmaceutical Sciences, Utrecht University, Utrecht, Netherlands; ^3^ Institute of Pharmacology and Toxicology, Rostock University Medical Center, Rostock, Germany

**Keywords:** transporters and channels, physiology, pharmacology, toxicology, pathophisiology

The emergence of the plasma membrane marked a pivotal milestone in the progression of cellular life. Alongside safeguarding genetic material, this structural development introduced a challenge: enabling the exchange of various substances—such as nutrients, foreign compounds (xenobiotics), hormones and metabolic by-products—between the interior and exterior compartments of the cell. To tackle this issue, the plasma membrane advanced through the integration of transport proteins facilitating the movement of charged hydrophilic elements across this membrane.

Transport proteins, constituting the most extensive family of membrane proteins within the human organism, are present in every cell. Consequently, these transporters hold profound significance in cellular physiology and exhibit a high degree of preservation throughout evolutionary processes. Across diverse organs, an array of transporters is integral, serving as fundamental determinants of their respective functionalities.

Transporters play a pivotal role in facilitating the absorption, distribution, metabolism, elimination, and toxicity (ADMET) of pharmaceutical compounds, influencing drug’s efficacy and safety.

Due to their involvement in these critical processes, transporters provide a pathway to explore crucial realms of physiology, toxicology, and pharmacology. Their significance extends as they offer insights into the intricate mechanisms governing drug interactions and responses within the body.

The TransportDays in Greifswald, Germany, place a spotlight on critical facets of transporter functionality. This event is tailored for and orchestrated by scientists passionate about the fields of physiology, pharmacology, and structural biology concerning cell membrane channels and transporters. These belong mostly to the ATP-Binding Cassette (ABC) to the solute carrier (SLC) transporter family (Thomas and Tampé, 2020; Pizzagalli et al., 2021).

Originally conceived by Prof. Gerhard Burckhardt to honor the legacy of Prof. Karl J. Ullrich, a trailblazer in renal transport physiology and the former director of the Max-Planck-Institute for Biophysics in Frankfurt (Murer and Burckhardt, 2010; Frömter and Schulz, 2011), the TransportDays initially drew participants primarily influenced by Prof. Ullrich. Over time, this gathering has evolved to encompass diverse groups actively engaged in transport physiology and pharmacology, both within and beyond Germany.

The thematic focus of this Research Topic, “*Transporters: a hitchhiker’s guide to physiology, toxicology, and pharmacology—TransportDays 2022*,” serves as a platform for publishing works stemming from the symposium and from researchers across these fields.

The review “*Role of transporters in regulating mammalian intracellular inorganic phosphate*” by Jennings provides an overview of the existing knowledge concerning the involvement of plasma membrane transporters in the regulation of intracellular inorganic phosphate [(Pi)In] in mammals. The influx of Pi is facilitated by Na^+^-Pi cotransporters, specifically SLC34 and SLC20, and counterbalanced by efflux through XPR1 (xenotropic and polytropic retrovirus receptor). Activity of these transporters is regulated by Pi concentrations.

The review “*Drug transporters in the kidney: Perspectives on species differences, disease status, and molecular docking*” by Zou et al. summarizes the knowledge on species, sex-genders, ages, and disease statuses influences on the renal expression of eleven drug transporters (*viz.* OAT1, OAT3, OATP4C1, OCT2, MDR1, BCRP, MATE1, MATE2-K, OAT4, MRP2, and MRP4) and on natural products that represent potential substrates and/or inhibitors of these transporters.

The review “*Cell surface transporters and novel drug developments*” by Carmichael and Day furnish evidence of the importance of plasma membrane transporters for drug ADMET studies. This review explores the development of plasma membrane transporter exploitation to increase drug specificity, reducing dosage and toxicity and thus revolutionising drug development.

The review “*Perfluorooctanoic acid (PFOA) exposure in relation to the kidneys: A review of current available literature*” by Liu et al. focuses on the mechanisms of renal toxicity induced by PFOA, an industrial by-product of manufacturing commercial polymers, which is an important component of protective coatings for textiles, leather, carpets and paper, of pesticides, paints and cosmetics, as well as of fire foam, hydraulic oil, wax and polishing agents. Current knowledge on renal PFOA handling by organic anion transporters in the tubular system is presented.

The original research work “*Regulation of ABC drug efflux transporters in human T-cells exposed to an HIV pseudotype*” by Whyte-Allmanet al. presents new findings indicating a potential link between the activation of the mTOR signaling pathway and the heightened expression of ABC drug efflux transporters in CD4^+^ T-cells when exposed to an HIV pseudotype. This increased expression of transporters might hinder the penetration of antiretroviral drugs into the T-cells targeted by HIV. Moreover, the presence of ABC transporters could potentially play a role in the secretion of proinflammatory cytokines associated with HIV infection.

The original research work “*Identification and characterization of a novel SNAT2 (SLC38A2) inhibitor reveals synergy with glucose transport inhibition in cancer cells*” by Gauthier-Coles et al. presents the identification of 3-(N-methyl (4-methylphenyl)sulfonamido)-N-(2-trifluoromethylbenzyl)thiophene-2-carboxamide as a potent inhibitor of SNAT2, which serves as a Na^+^-dependent neutral amino acid transporter. SNAT2 facilitates the accumulation of amino acids essential as nutrients, ensuring cellular osmolarity, and triggering the activation of mTORC1. Additionally, SNAT2 plays a role in supplying net glutamine for glutaminolysis, making it a potential target for cancer treatment.

The original research work “*Characterization of ligand-induced thermal stability of the human organic cation transporter 2 (OCT2)*” by Maane et al. presents a thermal shift assay (TSA) to analyse the thermodynamics governing OCT2 binding to different ligands. TSA can be useful in pinpointing substrates with great chemical complexity, which are probably characterized by higher solvation costs (high entropy).

The original research work “*Bile acid interactions with neurotransmitter transporters*” by Romanazzi et al. presents the effects of bile acids (BAs, a class of molecules synthesized by the liver that may also be present in the brain) and their relationship with substrates in transporters of the solute carrier 6 family. These results suggest a potential role of BAs as a treatment of neurodegenerative and neurological diseases.

The Guest Editors extend their sincere gratitude to all the authors and reviewers for their contributions to this Research Topic. Their dedication has been instrumental in shaping the quality of this publication. Additionally, the Guest Editors express their appreciation to the Frontiers Editorial Team for their excellent collaboration throughout this endeavour.
